# Integrated flavoromics and lipidomics analysis of metabolic difference and flavor regulation mechanisms in duck subcutaneous adipose tissue

**DOI:** 10.3389/fnut.2025.1671714

**Published:** 2025-09-16

**Authors:** Ping Gong, Shengqi Cheng, Xing Chen, Lixia Wang, Yanan Wang, Mingli Zhai, Yunguo Qian, Shengqiang Ye, Yu Yang

**Affiliations:** ^1^Animal Husbandry and Veterinary Research Institute, Wuhan Academy of Agricultural Sciences, Wuhan, China; ^2^College of Animal Science and Technology, Huazhong Agricultural University, Wuhan, China

**Keywords:** duck, subcutaneous adipose tissue, flavoromics, lipidomics, triglyceride

## Abstract

Subcutaneous adipose tissues (SAT) are critical determinants of duck meat flavor and texture. Investigating metabolic differences in SAT deposition facilitates targeted breeding strategies for superior flavor profiles and feed formulation optimization. This study employed flavoromics and lipidomics to systematically compare the flavoromics and lipidomics profiles of SAT from Wuqin-10 duck (WQ) and Cherry valley duck (CV). Flavoromics analysis identified hexadecanal as the sole significantly upregulated differential flavor compounds in WQ ducks (*P*-value < 0.05), predominantly enriched in fatty acid degradation pathways. Lipidomics revealed 182 differential lipids (87 upregulated, 95 downregulated) between breeds, with Carnitine C4:1-2OH exhibiting 256-fold higher relative abundance in WQ ducks. Compositional analysis demonstrated elevated levels of long-chain triglycerides (TAGs), lysophosphatidylcholines (LPCs), and lysophosphatidylethanolamines (LPEs) in WQ ducks, whereas CV ducks showed higher abundance of phosphatidylserine (PS), phosphatidylethanolamine (PE), and phosphatidylcholine (PC). These differential lipids were significantly enriched in glycerophospholipid metabolism, GPI-anchor biosynthesis, and polyunsaturated fatty acid metabolic pathways. Integrated multi-omics analysis further identified significant positive correlations between flavor compounds (n-hexadecanoic acid and hexadecanal) and glycerolipids (TG/DG). Collectively, this work systematically elucidates substantial compositional divergences in flavor compounds and lipids between CV and WQ ducks, while flavor compound-lipid correlations reveal potential regulatory nodes within lipid metabolic networks, providing crucial molecular insights into breed-specific lipid metabolic characteristics.

## 1 Introduction

The subcutaneous adipose tissue (SAT) of duck plays a central role in duck meat quality formation, wherein its abundant lipids undergo complex thermally induced oxidative reactions during cooking, generating key volatile organic compounds-including aldehydes, ketones, alcohols, and esters-that constitute the characteristic aroma profile of duck SAT (1). For instance, oxidative degradation of unsaturated fatty acids yields aldehyde and ketone compounds imparting the distinctive lipid-derived aroma to duck meat (2, 3). Concurrently, melted SAT permeates muscle tissues during cooking, significantly enhancing water-holding capacity and tenderness while synergistically contributing to its unique aroma and flavor profile (1, 4, 5).

The Wuqin-10 duck (WQ duck) is a novel high-quality meat duck variety developed through modern breeding techniques targeting key economic traits including plumage color, SAT deposition rate, and reproductive performance. This strain exhibits distinguishing characteristics such as high lean meat yield (up to 27.5%) and relatively low SAT deposition rate, coupled with superior meat texture and flavor profiles, establishing it as an ideal raw material for value-added duck processing (3). In contrast, the Cherry Valley duck (CV duck)-a prominent meat duck breed-is renowned for its rich fatty acid composition, predominantly featuring monounsaturated fatty acids (MUFAs, e.g., oleic acid/C18:1 n-9) and saturated fatty acids (SFAs), alongside moderate levels of polyunsaturated fatty acids (PUFAs, e.g., linoleic acid/C18:2 n-6) compared with Grimaud duck (6). These fatty acids critically sustain avian physiological functions and contribute to breed-specific meat flavor development (6–8). Crucially, the elevated lipid content and distinctive fatty acid profile-particularly the proportion of unsaturated fatty acids-enable CV duck SAT to undergo extensive oxidation and degradation during cooking (especially roasting), generating abundant volatile flavor compounds (aldehydes, ketones, esters, etc.) that collectively form its signature rich, meaty aroma profile (2, 9).

Consequently, SAT quality constitutes a pivotal determinant of the ultimate eating quality (texture, flavor, nutritional value) of duck meat products (10). Nevertheless, the specific divergences in metabolic signatures (metabolome) and lipid molecular species/composition (lipidome) across duck breeds, along with their regulatory mechanisms, remain inadequately elucidated. The Cherry Valley duck is renowned as a fast-growing meat-type breed, characterized by its rapid growth and high subcutaneous and abdominal fat deposition, whereas the WQ duck, as a high-quality meat duck hybrid line independently developed in China, achieves a balance between growth rate and meat quality traits. We employed integrated flavoromics and lipidomics to systematically compare their flavor compounds and lipids profiles. This investigation not only contributes to elucidating the mechanisms of lipid metabolism and identifying key biomarkers, but also provides a theoretical basis for breeding meat duck breeds that better align with the flavor preferences and processing requirements of the Chinese market.

## 2 Materials and methods

### 2.1 Experimental design and animal management

Three-day-old Cherry Valley (CV) and Wuqin-10 (WQ) ducklings (*n* = 120 per breed) with health and comparable body weights were selected. Ducklings were divided into two groups (CV and WQ), with each group containing 12 replicates of 10 ducklings (with equal sex distribution). Identical husbandry management practices were applied to both groups. Ducks had ad libitum access to feed and water, and no antibiotics were administered during the month preceding slaughter.

### 2.2 Sample collection

At 63 days of age, one duck per replicate was randomly selected (*n* = 12) and fasted for 8 hours. Following slaughter, SAT were immediately collected and snap-frozen in liquid nitrogen for subsequent omics analyses.

### 2.3 Flavoromics

#### 2.3.1 Sample preparation

Headspace solid-phase microextraction (HS-SPME): Samples retrieved from-80 °C were pulverized in liquid nitrogen and vortex-homogenized. Approximately 1g SAT was weighed into headspace vials, followed by addition of 1-2 mL saturated NaCl solution for single-step ionic strength/pH adjustment and 10 μL internal standard solution. The SPME fiber assembly was exposed to the vial headspace for volatile flavor compound adsorption under temperature/time-specific conditions. Simultaneous distillation-extraction: Sample-water mixtures were loaded into one chamber of the distillation apparatus while organic solvent was placed in the opposing chamber. Concurrent distillation and extraction were performed, whereby volatile flavor compounds migrated with steam into the organic phase under thermal convection and condensation forces. After timed extraction, the organic phase was collected and concentrated via nitrogen-blowing.

#### 2.3.2 Chromatography and mass spectrometry conditions

Chromatographic conditions: The SPME injection parameters included an aging temperature of 250 °C for 5 min, heating at 60 °C for 10 min, adsorption time of 20 min, desorption time of 5 min, and post-injection re-aging time of 5 min. Mass spectrometry conditions: electron bombardment ionization source with 70 eV electron energy; scan range typically set to m/z 35–550 to cover fragment ions of most flavor compounds; interface temperature maintained at approximately 280 °C to prevent condensation or decomposition during sample transfer. For sample analysis: the extracted flavor compounds were injected into the GC-MS system to initiate the analytical program for automated separation and detection. Each sample was typically analyzed 2–3 times with averaged values taken to ensure data accuracy. The internal standard was [2H7]-2-methylpyridine, and the chromatographic column was DB-5MS (30 m x 0.25 mm, 0.25 μm).

#### 2.3.3 Data processing and analysis

Data acquisition: Data acquisition was performed using a GC-MS system, which generated total ion chromatograms (TIC) by recording ion intensity over time. Both chromatographic and mass spectrometric data were saved using the data acquisition software for subsequent processing. Peak identification and integration: Chromatographic peaks within the TIC were identified and integrated using GC-MS data analysis software to determine their retention times and peak areas. The peak area was used as a measure of the relative abundance of each flavor compound. Qualitative analysis: Mass spectral fragmentation patterns were compared against standard reference libraries (e.g., the NIST database). Compound identities were confirmed based on matching scores and retention times. Flavor compounds with a fold change ≥2 or fold change ≤ 0.5, *P*-value < 0.05 and VIP ≥ 1 were selected.

### 2.4 Lipidomics

#### 2.4.1 Sample preparation

Reference to Folch method for lipid extraction (11): Approximately 1 g of SAT sample was weighed, and a methanol–chloroform mixture (2:1, v/v) was added at a ratio of 1:10 (w/v). The mixture was homogenized at low temperature. The homogenate was then transferred to a separatory funnel, followed by the addition of 1/5 volume of 0.9% sodium chloride solution. The mixture was shaken vigorously and allowed to separate into phases. The lower organic phase was collected, dehydrated with anhydrous sodium sulfate, and filtered. The filtrate was concentrated by rotary evaporation and finally dried under a nitrogen stream to obtain the lipid extract.

#### 2.4.2 Lipid separation and detection

Lipid Separation by Reversed-Phase Liquid Chromatography (RPLC): This technique employed a non-polar stationary phase and a polar mobile phase. The non-polar moiety of lipids interacted with the stationary phase while the polar moiety interacts with the mobile phase. Lipids with stronger non-polar characteristics exhibited greater retention on the stationary phase; as the polarity of the mobile phase was increased, lipids were sequentially eluted in decreasing order of hydrophobicity. Lipid Detection via Mass spectrometry: Mass spectrometry was used to ionize lipid molecules and separate/detect ions based on their mass-to-charge ratios (m/z). In the ion source, lipid molecules absorbed energy to form ions by gaining or losing electrons. These ions were spatially or temporally resolved under electric and magnetic fields according to their m/z values, ultimately being detected to generate mass spectra. Distinctive m/z signatures and characteristic fragment ions of different lipids enabled qualitative and quantitative analysis through comparison with standard spectral libraries. The internal standard were [12:0 Lyso PC, Cer (d18:1/4:0), PC (13:0/13:0), DG (12:0/12:0), TG (17:0/17:0/17:0)], and the chromatographic column was (Thermo Accucore™ C30 column, i.d. 2.1 × 100 mm, 2.6 μm).

#### 2.4.3 Data processing and analysis

Raw data processing: The acquired raw data underwent processing, including noise removal, baseline correction, peak detection, and integration to enhance data quality. Lipid identification was achieved by comparing retention times and mass spectra with authentic standards, while quantification was performed based on peak areas or signal intensities. Database searches and specialized software were used to further facilitate lipid annotation and quantitation. These lipids were functionally annotated by mapping to biological metabolic pathways, revealing their involvement in specific biological processes and signaling cascades. Pathway enrichment analysis (e.g., using KEGG database) elucidated the biological implications of lipid alterations. The screening criteria for differential lipids are consistent with flavoromics.

### 2.5 Correlation analysis between flavoromics and lipidomics

The correlation analysis (RDA redundancy analysis) between metabolome and lipidomics was performed by the genescloud tools, a free online platform for data analysis (https://www.genescloud.cn).

### 2.6 Data statistical analysis

The data were analyzed by Mann-Whitney *U* test in SPSS 24.0. ^*^P-value < 0.05 indicates a significant difference, ^**^P-value < 0.01 indicates an extremely significant difference, and NS (*P*-value > 0.05) indicates that there is no significant difference between the data.

## 3 Results

### 3.1 Flavoromics quality control analysis

**Figure 1:**
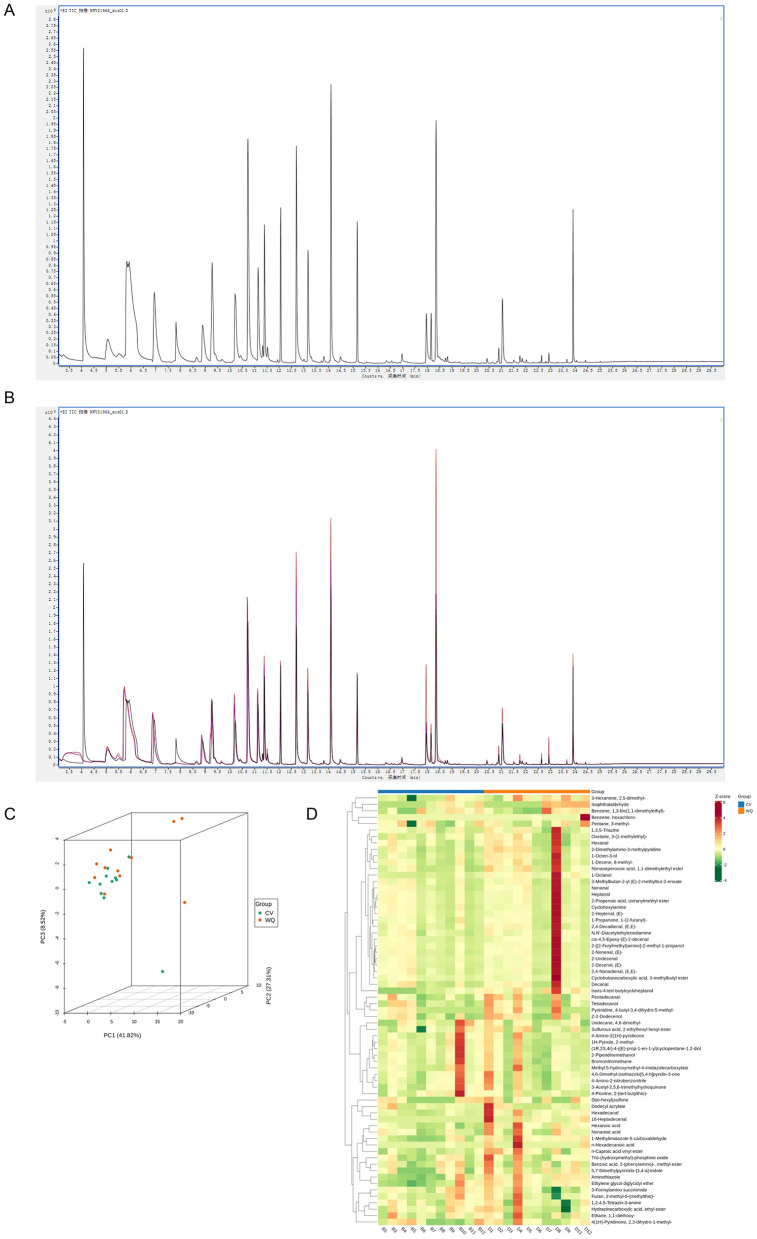
Quality control assessment of flavoromics raw data. **(A)** Flavor compounds qualitative and quantitative analysis. **(B)** Quality control analysis of samples. Overlaid total ion chromatograms (TIC) from mass spectrometry of QC samples. The high overlap of flavor compound detection TIC curves indicates consistent retention time and peak intensity, demonstrating good signal stability across repeated instrument analyses of the same sample. The high instrument stability ensures data repeatability and reliability. **(C)** Principal component analysis (PCA) of samples. **(D)** Hierarchical cluster analysis of samples. *X*-axis: Sample names. *Y*-axis: All flavor compounds. Red: High abundance; Green: Low abundance.

In this study, the proportion of peaks with CV ≤ 30% in QC samples to the total number of peaks are over 85% (Supplementary Figure S1), indicating that the instrumental analysis system has good stability and the data can be used for subsequent analysis. Following qualitative analysis of raw instrument data, Figure 1A displays the total ion chromatogram (TIC) of pooled quality control (QC) samples, representing the continuous plot of summed ion intensities at each time point. Flavor compound identification and quantification were performed using the NIST database, detecting 67 flavor compounds (Supplementary Table S1) with their respective names and relative abundances. The QC samples, prepared by pooling random specimens, assessed technical reproducibility under identical processing conditions. Results demonstrated excellent repeatability in flavor compound extraction and detection, confirming data reliability (Figure 1B). Principal Component Analysis (PCA) of all samples revealed distinct clustering patterns among groups, indicating inter-group flavoromics differences (After removing outlier sample, *n* = 11) (Figure 1C). Unit Variance (UV)-scaled flavor compound abundance data underwent hierarchical cluster analysis (HCA) via the R pheatmap package. Heatmap visualization demonstrated group-specific accumulation patterns, exemplified by higher relative abundances of hexanoic acid, non-anoic acid, n-hexadecanoic acid, and n-caproic acid vinyl ester in the WQ group vs. the CV group (Figure 1D).

### 3.2 Identification of differential flavor compounds and KEGG enrichment analysis

**Figure 2:**
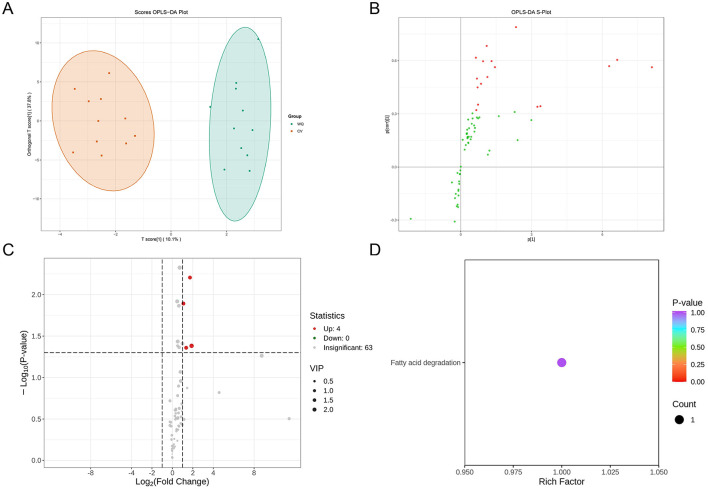
Screening and identification of differential flavor compounds. **(A)** OPLS-DA score plot. **(B)** OPLS-DA S-Plot. **(C)** Screening of differential flavor compounds. Each point in the volcano plot represents a flavor compound. *X*-axis: Log_2_foldchange in abundance between two sample groups. *Y*-axis: VIP (Variable Importance in Projection) value. Larger absolute values on the *X*-axis indicate greater fold changes. Higher *Y*-axis values indicate more statistically significant differences. Green points: Down-regulated flavor compounds; Red points: Up-regulated flavor compounds; Gray points: Flavor compounds detected but not significantly different. **(D)** KEGG functional annotation and pathway enrichment analysis of differential flavor compounds. *X*-axis: Rich Factor for each pathway. *Y*-axis: Pathway names (sorted by *P*-value). Point color reflects *P*-value (redder indicates higher significance). Point size represents the number of enriched differential flavor compounds.

PLS-DA score plot visualization revealed distinct separation between CV and WQ duck SAT samples (each point representing an individual specimen), indicating systemic flavoromics differences between breeds. Both groups exhibited intra-group clustering tendencies while demonstrating within-group variations (Figure 2A). In the OPLS-DA S-plot (where each point denotes a flavor compound), flavor compounds in the upper-right quadrant showed significant up-regulation in WQ ducks with high model contribution, whereas lower-left quadrant flavor compounds were markedly down-regulated in WQ ducks with strong discriminatory power. This plot confirmed discriminant flavor compounds driving inter-breed differences and indicated their abundance trends (Figure 2B). Volcano plot analysis identified 4 up-regulated flavor compounds (hexadecanal (palmitic aldehyde), hexanoic acid, 1-Octanol and trans-4-tert-butylcycloheptanol) in WQ vs. CV ducks (foldchange > 2, *P*-value < 0.05), with zero down-regulated and 63 non-significant flavor compounds (Figure 2C). KEGG pathway enrichment analysis of this differential flavor compounds exclusively highlighted the fatty acid degradation pathway (Figure 2D).

### 3.3 Lipidomics quality control analysis

**Figure 3:**
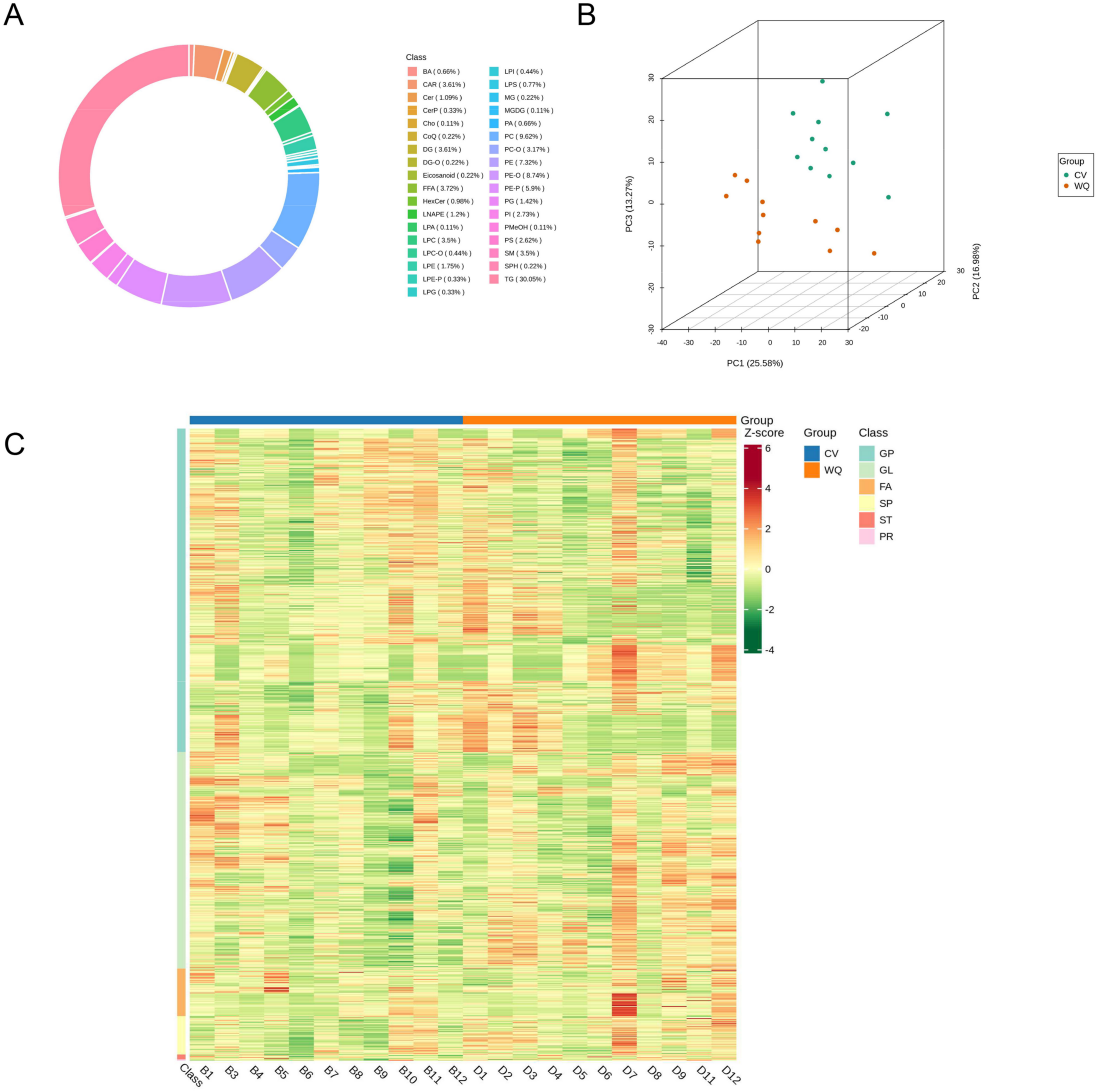
Quality control assessment of lipidomics raw data. **(A)** Circular plot of lipid subclass composition. **(B)** Principal component analysis (PCA) of samples. **(C)** Hierarchical cluster analysis of samples.

Lipidomics profiling identified 915 lipid species in total (Supplementary Table S2). Lipid composition exhibited sample-specific patterns with distinct subclass distributions across sample types. Specifically, duck SAT comprised 30.05% triglycerides (TG), 9.62% phosphatidylcholines (PC), 8.74% ether-linked phosphatidylethanolamines (PE-O), and 5.90% plasmalogen phosphatidylethanolamines (PE-P) (Figure 3A). Principal Component Analysis (PCA) of all samples (including QCs) demonstrated group separation trajectories, suggesting inter-group lipidomics divergence (After removing outlier sample, *n* = 11) (Figure 3B). Following unit variance scaling normalization of lipid abundance data, hierarchical cluster analysis (HCA) was performed using R software (https://www.r-project.org/) to examine accumulation patterns. Clustering profiles differed significantly between groups: PS, PE, and PC lipids predominated in CV ducks, whereas long-chain TGs, lysophosphatidylcholines (LPC), and lysophosphatidylethanolamines (LPE) were enriched in WQ ducks (Figure 3C).

### 3.4 Identification of differential lipid and KEGG enrichment analysis

**Figure 4:**
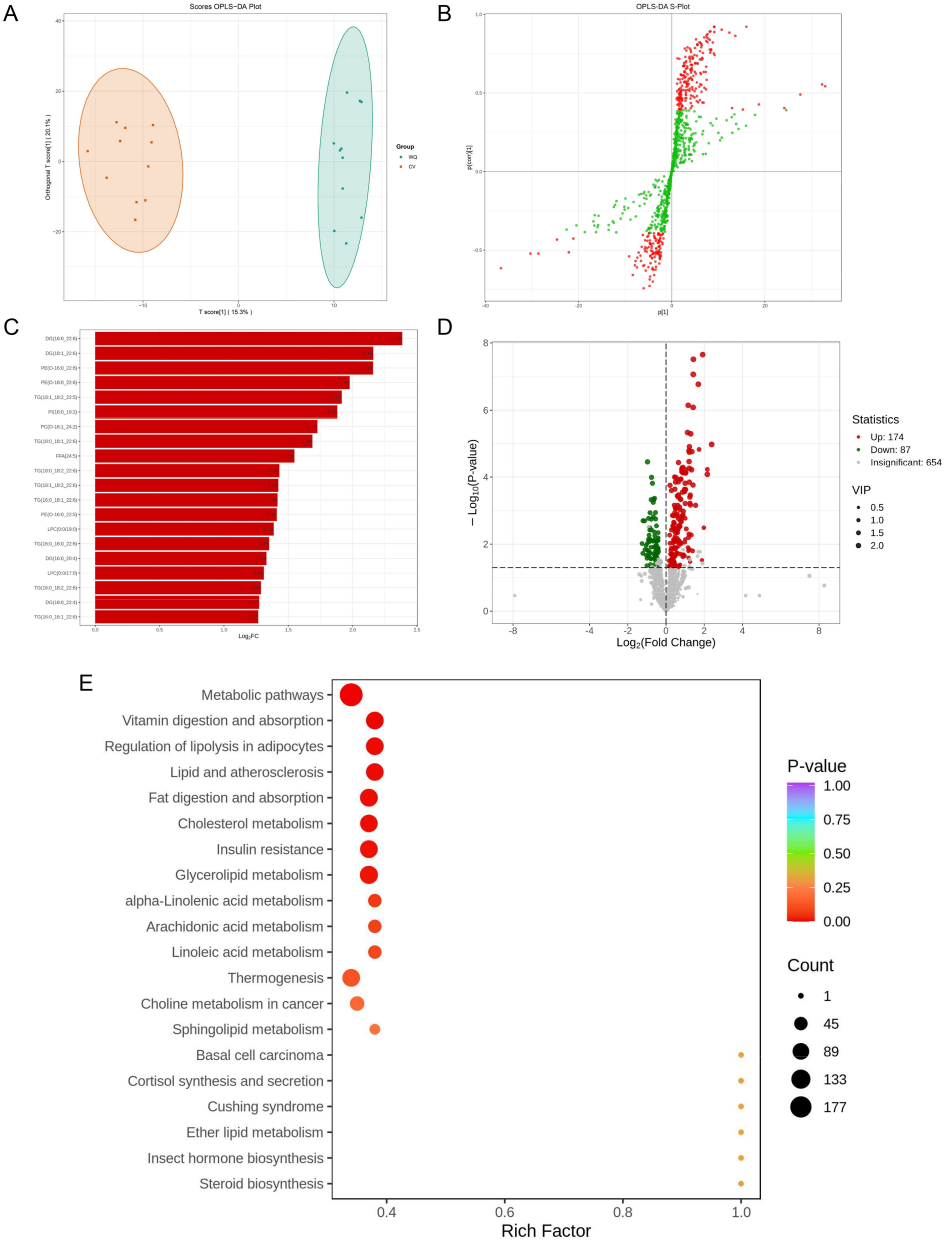
Screening and identification of differential lipids. **(A)** OPLS-DA score plot. **(B)** OPLS-DA S-Plot. **(C)** Bar plot of differential lipids. *X*-axis: Log_2_FC (Log_2_Fold Change) of differential lipids. *Y*-axis: Differential lipids. Red: Up-regulated lipids; Green: Down-regulated lipids). **(D)** Volcano plot of differential lipids. **(E)** KEGG functional annotation and pathway enrichment analysis of differential lipids.

PLS-DA analysis revealed pronounced spatial separation between WQ and CV duck SAT samples (Figure 4A), demonstrating the model's discriminative capacity to distinguish groups based on lipidomics profiles. This confirms significant systemic differences in SAT metabolism between breeds. The OPLS-DA S-plot highlighted core differential flavor compounds (red points in upper-right/lower-left quadrants) with strong discriminatory power and high correlation to group separation (Figure 4B). These flavor compounds potentially involved in subcutaneous adipose tissue synthesis, degradation, and transport pathways-represent key drivers of inter-breed metabolic divergence. Subsequently, we identified the top 20 most differentially abundant lipids (|log_2_FC| > 1, *P*-value < 0.05). Notably, 19 core lipids were significantly upregulated in WQ ducks, with carnitine C4:1-2OH exhibiting 256-fold enrichment (log_2_FC = 8), indicating enhanced subcutaneous adipose tissue metabolic activity (Figure 4C). Volcano plot analysis detected 261 differential lipids between breeds: 174 upregulated and 87 downregulated in WQ vs. CV ducks, alongside 654 non-significant lipids (Figure 4D). KEGG enrichment analysis of differential lipids revealed predominant involvement in: regulation of lipolysis in adipocytes, glycerolipid metabolism, α-linolenic acid metabolism, arachidonic acid metabolism and linoleic acid metabolism (Figure 4E).

### 3.5 Correlation analysis between metabolome and lipidomics

**Figure 5:**
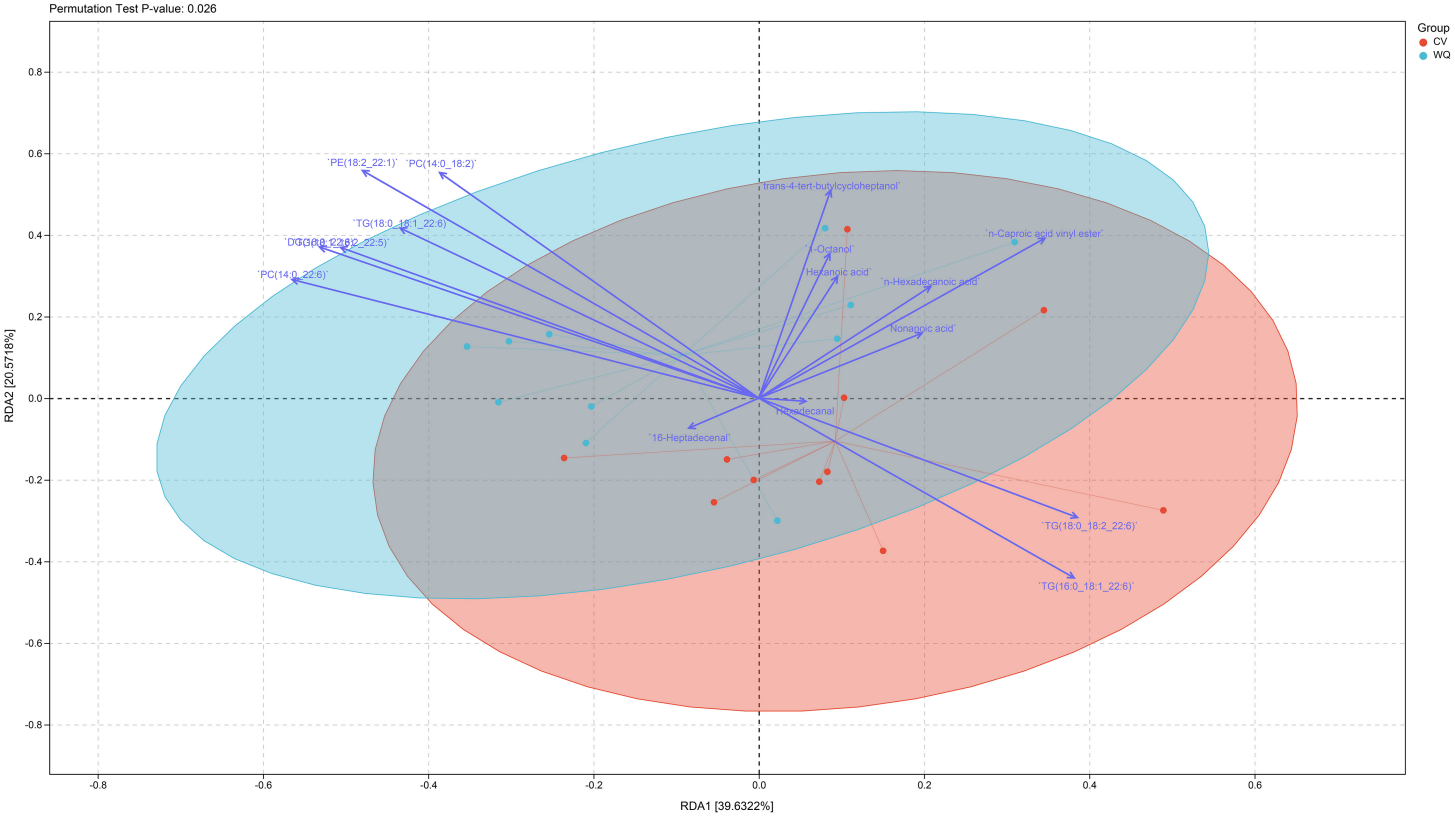
Correlation analysis of metabolome and lipidome data.

Integrated flavoromics and lipidomics analyses revealed a highly significant difference (*P*-value = 0.026) in the overall flavoromics and lipidomics profiles between WQ ducks and CV ducks. Furthermore, significant positive correlations were observed in duck SAT between flavor compounds (n-hexadecanoic acid, hexanoic acid, non-anoic acid, hexadecanal) and lipids (TGs) (Figure 5).

## 4 Discussion

The distinct flavor profiles of duck sebum critically influence consumer preferences, yet the molecular regulatory mechanisms remain elusive due to metabolic network complexity. Substantial evidence confirms that characteristic flavor compounds derive primarily from lipoxygenase-catalyzed or autoxidative decomposition of polyunsaturated fatty acids (12, 13), directly linking volatile flavor compounds and fatty acid composition to flavor formation (14, 15). Integrating untargeted flavoromics and lipidomics, this study systematically deciphered the molecular basis and pathway-driven mechanisms underlying flavor differences between CV and WQ duck sebum.

Flavoromics analysis revealed pronounced oxidative metabolism in WQ duck sebum, where hexadecanal (palmitic aldehyde), hexanoic acid, 1-Octanol and trans-4-tert-butylcycloheptanol-the significantly upregulated differential flavor compounds (Figure 2C) enriched in fatty acid degradation pathways-directly reflects accumulated lipid oxidation end-products (16). As a characteristic β-oxidation product of n-hexadecanoic acid (17, 18), it imparts grassy and waxy undertones, molecularly explaining WQ's intensified lipidic aroma (19, 20). Notably, aldehydes serve as core flavor contributors: hexanal (linoleate oxidation) presents fresh-green notes; octanal and non-anal (oleate-derived) contribute fruity/fatty and floral/fatty aromas, respectively (21, 22). Medium-chain fatty acids (hexanoic/non-anoic acid) directly participate in flavor perception while serving as aldehyde/ketone precursors (23, 24). Quality control data and clustering patterns collectively validated metabolic reliability, indicating active lipolysis-oxidation equilibrium in WQ duck SAT.

Lipidomics highlighted fundamental inter-breed differences: CV ducks prioritized structural phospholipids (PS/PE/PC) maintaining membrane integrity (25–27), whereas WQ ducks significantly enriched long-chain triglycerides (TG) and lysophospholipids (LPC/LPE), establishing “hydrolysis-oxidation coupled dynamics” that liberate unsaturated free fatty acids for flavor transformation (28, 29). Thermal oxidation of these unsaturated fatty acids generates peroxides decomposing into flavor-critical volatile compounds (2, 30, 31). This metabolic landscape was reinforced by Carnitine C4:1-2OH (sic), which transports free fatty acids (e.g., oleic/palmitic acid) to mitochondria for β-oxidation (reducing greasiness) (32), while scavenging free radicals to suppress rancid aldehyde generation (33). KEGG enrichment further corroborated activation of glycerophospholipid metabolism (membrane hydrolysis) and arachidonic acid metabolism (oxidative stress response) jointly provisioning flavor precursors (34, 35).

Integrated analysis established a cascade mechanism: TG hydrolysis releases n-hexadecanoic acid that undergoes β-oxidation into hexadecanal, contributing foundational “unctuousness” to heated duck skin though excess causes unpleasant odors (36, 37). Hexadecanal's exclusive enrichment in fatty acid degradation pathways forms a WQ-specific “high TG hydrolysis-FFA accumulation- elevated aldehydes” cascade shaping robust aroma (2). Conversely, CV ducks' structural lipid dominance likely constrains TG hydrolysis efficiency. This synergy between lipid composition (substrate reservoir) and enzyme activity (conversion capacity) drives flavor divergence, while aldehyde accumulation thresholds warrant vigilance against rancidity in WQ ducks (33).

The gut microbiota plays a central role as a “metabolic regulator” in the deposition of subcutaneous fat in ducks, extending far beyond auxiliary digestion to deeply participate in the process through multiple mechanisms. By fermenting dietary fibers that are difficult for ducks to digest on their own, gut microbes produce short-chain fatty acids (SCFAs, such as acetate, propionate, and butyrate). These SCFAs serve not only as direct substrates for fat synthesis and are transported to adipose tissue but also act as signaling molecules that activate or inhibit host metabolic pathways—including the AMPK pathway—thereby regulating the balance between fat synthesis and breakdown and ultimately influencing the efficiency of subcutaneous fat deposition. In the future, we plan to investigate the gut microbiota–liver–subcutaneous fat axis in ducks. A deeper understanding of the mechanisms behind this axis holds significant theoretical and practical implications for optimizing duck production performance, health status, and product quality-particularly fat content and composition-through nutritional interventions such as modulating microbial communities.

## 5 Conclusion

In this study, although the number of differential flavor compounds detected by flavoromics was limited (hexadecanal (palmitic aldehyde), hexanoic acid, 1-octanol, and trans-4-tert-butylcycloheptanol), lipidomics analysis revealed significant enrichment of Carmirine C4:1-2OH and long-chain TGs in the SAT of WQ ducks. These differential molecules primarily involved key pathways such as fatty acid degradation, glycerophospholipid metabolism, and unsaturated fatty acid metabolism. Strong correlations between flavor compounds and lipids further suggested potential regulatory nodes within the SAT metabolic network. These findings provide critical insights for understanding the metabolic characteristics and molecular basis of SAT across duck breeds.

## Data Availability

The original contributions presented in the study are included in the article/Supplementary material, further inquiries can be directed to the corresponding authors.

## Supplementary material

Supplementary Figure S1 Distribution Chart of QC Sample Coefficient of Variation. **(A)** Distribution of coefficient of variation of QC samples in flavoromics. **(B)** Distribution of coefficient of variation of QC samples in lipidomics.

Supplementary Table S1 Flavoromics data of subcutaneous adipose tissue from WQ duck and CV duck.

Supplementary Table S2 Lipidomics data of subcutaneous adipose tissue from WQ duck and CV duck.
