# Supplementary figures and images for: Integrated flavoromics and lipidomics analysis of metabolic difference and flavor regulation mechanisms in duck subcutaneous adipose tissue
Source: Front Nutr. 2025 Sep 16;12:1671714. doi: 10.3389/fnut.2025.1671714 (PMC12479485; doi:10.3389/fnut.2025.1671714)

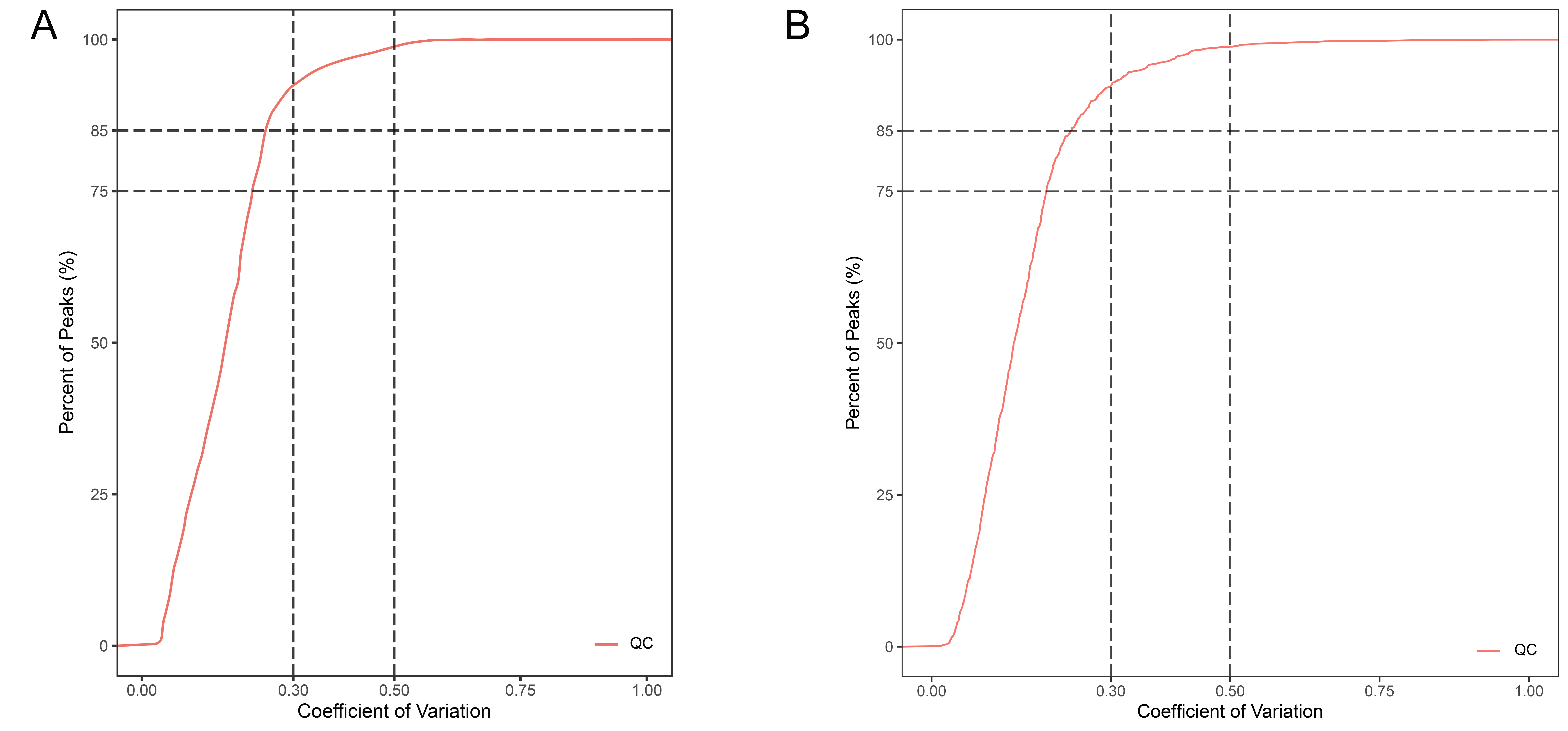

Supplement: Supplementary Figure S1 — Distribution Chart of QC Sample Coefficient of Variation. (A) Distribution of coefficient of variation of QC samples in flavoromics. (B) Distribution of coefficient of variation of QC samples in lipidomics. [file Image_1.tif]
